# Static and Dynamic Behavior of Polymer/Graphite Oxide Nanocomposites before and after Thermal Reduction

**DOI:** 10.3390/polym13071008

**Published:** 2021-03-25

**Authors:** Kiriaki Chrissopoulou, Krystalenia Androulaki, Massimiliano Labardi, Spiros H. Anastasiadis

**Affiliations:** 1 Institute of Electronic Structure and Laser, Foundation for Research and Technology-Hellas, N. Plastira 100, 700 13 Heraklion Crete, Greece; krysand@iesl.forth.gr (K.A.); spiros@iesl.forth.gr (S.H.A.); 2 Department of Chemistry, University of Crete, P.O. Box 2208, 710 03 Heraklion Crete, Greece; 3 CNR-IPCF, c/o Physics Department, University of Pisa, Largo Pontecorvo 3, 56127 Pisa, Italy; labardi@df.unipi.it

**Keywords:** hyperbranched polymers, graphite oxide, intercalation, dynamics, thermal reduction, reduced graphite oxide, conductivity

## Abstract

Nanocomposites of hyperbranched polymers with graphitic materials are investigated with respect to their structure and thermal properties as well as the dynamics of the polymer probing the effect of the different intercalated or exfoliated structure. Three generations of hyperbranched polyester polyols are mixed with graphite oxide (GO) and the favorable interactions between the polymers and the solid surfaces lead to intercalated structure. The thermal transitions of the confined chains are suppressed, whereas their dynamics show similarities and differences with the dynamics of the neat polymers. The three relaxation processes observed for the neat polymers are observed in the nanohybrids as well, but with different temperature dependencies. Thermal reduction of the graphite oxide in the presence of the polymer to produce reduced graphite oxide (rGO) reveals an increase in the reduction temperature, which is accompanied by decreased thermal stability of the polymer. The de-oxygenation of the graphite oxide leads to the destruction of the intercalated structure and to the dispersion of the rGO layers within the polymeric matrix because of the modification of the interactions between the polymer chains and the surfaces. A significant increase in the conductivity of the resulting nanocomposites, in comparison to both the polymers and the intercalated nanohybrids, indicates the formation of a percolated rGO network.

## 1. Introduction

Nanohybrids composed of polymers and different types of additives, which have at least one dimension in the nm range, have attracted scientific interest since they usually possess superior properties compared to neat polymers or to composites that contain large reinforcing moieties [1,2,3,4,5,6,7,8,9]. Examples of such nanoadditives are certain layered materials like clays, graphene-based and/or other 2-D materials, as well as nanoparticles like silicon oxide (SiO_2_) and titanium oxide (TiO_2_) among others, or tubular materials like carbon nanotubes. One of the most important prerequisites for the utilization of such nanocomposites for the design of innovative materials that could be candidates for advanced applications is the understanding of their structure–dynamics-properties relation [10,11,12,13,14,15,16]. The way polymer structure and morphology as well as chain conformations are altered in the proximity of the additive surfaces and/or under confinement has been the subject of many investigations since these are among the parameters that define the final properties of such materials; their modification can act synergistically with the reinforcement offered by the nanoparticles [17,18,19,20,21,22,23,24]. At the same time, exploring the dynamic response and identifying the relaxation processes of the polymers in the nanocomposites is of vital importance since it is the dynamics that define the processability of these materials [25,26,27,28,29,30].

The key parameter for developing a nanocomposite with promising properties and, at the same time, one of the most important experimental challenges is the fine dispersion of the additive within the polymeric matrix. This strongly depends on the polymer/surface interactions and, thus, a great effort has been devoted towards both understanding and/or modifying these interactions [31,32]. This is even more critical in the case of layered additives, in which the interactions not only affect the dispersion of the additive within the polymer matrix, but they define the final nanohybrid structure as well. In such cases, three different structures can be obtained depending on the interactions [33,34]: (i) the phase separated, when the interactions between the chains and the fillers are unfavorable, which results in each component residing in its own phase; (ii) the intercalated one [17,28,29,35,36,37], where the polymer can diffuse between the layers of the additive, creating a structure of alternating soft–hard components, each one of nm-thickness, and (iii) the exfoliated one [38,39,40,41,42,43], where the favorable interactions lead to the destruction of the layered structure of the inorganic material and to the dispersion of individual platelets within the polymer. Both intercalated and exfoliated structures offer certain advantages: the former shows enhanced mechanical properties, while for hybrids with low polymer content, it offers the ability to investigate polymer structure, dynamics and properties under severe confinement, whereas the latter provides optimized properties due to the percolated network formed from the dispersion of the additive platelets [44,45]. Nevertheless, the opportunity to obtain both structures with the same materials, so that one can compare the polymer properties and dynamics, is only seldom achieved.

An interesting class of multilayered materials is the graphitic materials, like graphite itself or graphite oxide. In recent years, the most popular member of this group has been graphene, which is the single layer graphitic material utilized for the preparation of polymer nanocomposites because of its excellent properties [46,47,48,49]. Although it is mainly graphene or reduced graphene oxide that have been utilized for the preparation of polymer nanocomposites, there are a few works that employed graphite oxide as well [50,51,52,53]. Nevertheless, only in a few of them did the authors investigate the structure and dynamics of polymers under confinement [27,54,55,56]. Graphite oxide is hydrophilic, due to its various functional groups resulting from the oxidation of graphite, and, thus, it can be mixed with hydrophilic polymers to obtain nanocomposites. Moreover, its in situ thermally induced reduction in the presence of the polymer, i.e., the removal of functional hydrophilic groups, affects the interactions with the polymer and may even lead to a change in the structure of the nanocomposite, with a subsequent change of its properties. 

Polymer architecture is known to affect the properties of a polymer, such as the size of its chains, its solubility in different solvents, its glass transition temperature as well as its viscosity and the different kinds of relaxation processes. A class of non-linear polymers that have attracted scientific interest for their potential applications are the dendritic ones [57,58] which can be divided into dendrimers and hyperbranched polymers (HBPs). Both types of polymers are characterized by their generation number and the existence of multiple functional groups in their periphery. On the other hand, they differ in the perfectly symmetric structure and the well-defined molecular weight of the former versus the asymmetric structure and a certain degree of polydispersity of the latter. An additional advantage of HBPs is their cost-effective synthesis, and preservation of the beneficial properties of the dendritic materials [59]. Among the various HBPs, hyperbranched polyesters have been investigated mostly regarding their synthesis and their molecular characterization [60,61,62] and less regarding their structure and their ability for hydrogen-bond networks formation [63], their structure–property relationship [64] and their dynamics [28,65,66,67,68,69].

Hyperbranched polyesters have been utilized in the past to functionalize the surface of graphite oxide [70]. In a recent work [71], a hyperbranched polyester amide was used to develop nanocomposites with both graphite oxide and sodium montmorillonite clay and to investigate the effect of the different interfacial interactions on the structure of the nanohybrids, their thermal properties and on the resulting polymer dynamics. In both systems, the favorable interactions led to intercalated structures; nevertheless, polymer nanofilms of different thicknesses between the inorganic layers were obtained due to the different interaction strengths. In both cases, the glass transition of the intercalated chains was fully suppressed, whereas the different surface interactions affected polymer dynamics by modifying the number of relaxation processes as well as their relaxation times and their temperature dependencies. 

In the present work, hyperbranched polyester polyols were mixed with graphite oxide to develop nanocomposites, and investigate their structure, thermal properties and dynamic response before and after the thermally induced reduction of the nanoadditive and the de-oxygenation of its layers, which modifies the polymer/surface interactions. When the polymers are mixed with the layered hydrophilic graphite oxide, the favorable interactions lead to an intercalated structure. The nanohybrid composition studied was chosen so that all polymer chains reside within the galleries of the additive. For these systems, differential scanning calorimetry did not show any thermal transition associated with the confined chains. Dielectric relaxation spectroscopy was utilized to investigate the dynamics of the confined chains and it showed the existence of two local relaxations at temperatures below the bulk polymer glass transition temperature, which are attributed to the hydroxyl rotation and the ester reorientation with significantly lower activation energies than in the bulk due to the decreased ability to form inter- and intra-molecular hydrogen bonds under confinement. The segmental relaxation is observed as well, however, it exhibits an Arrhenius temperature dependence. Thermal reduction in the presence of the polymer indicates differences in the reduction mechanism, since a significant increase in the de-oxygenation temperature is observed. Following the reduction of graphite oxide (GO), the intercalated structure of the original nanohybrid is destroyed, and the dispersion of the additive layers in the matrix leads to an increase in the conductivity of the nanohybrid because of the formation of a percolated network by the dispersed platelets.

## 2. Materials and Methods

### 2.1. Materials

**Scheme 1:**
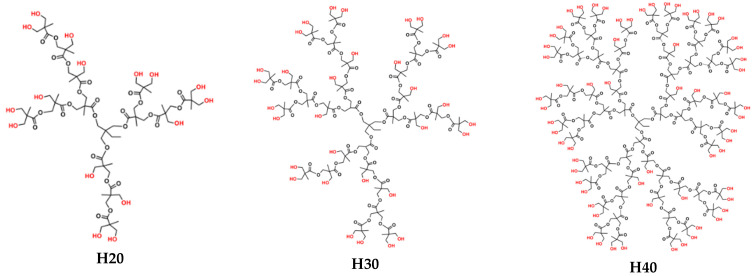
The three generations of the hyperbranched polyester polyols Boltorn^TN^.

Three commercial hyperbranched polyester polyols (Scheme 1) that were supplied by Polymer Factory were utilized in the current work. They are the second (H20), third (H30), and fourth (H40) pseudo-generations of the Boltorn Premium Series hyperbranched polymers with average molecular weights of 1750, 3608 and 7323 g/mol, respectively; their names are Boltorn^TN^ Premium H20, Boltorn^TN^ Premium H30 and Boltorn^TN^ Premium H40 (https://www.polymerfactory.com/boltorn, accessed on 2 March 2021). Their branches consist mainly of ester groups and possess an increasing number of hydroxyl end-groups with increasing generation (i.e., an average of 16 for H20, 32 for H30 and 64 for H40). It is these functional groups that make the polymers highly polar and hydrophilic and, thus, soluble in water. At the same time, they render the molecules capable of forming both intra- and inter-molecular hydrogen bonds between the hydroxyl groups and the oxygens of the ester groups.

The graphite oxide utilized was purchased from ACS Material (CAS No 7782-42-5 https://www.acsmaterial.com/graphite-oxide.html, accessed on 2 March 2021) and was produced by the Hummer’s method with a lateral size of ~0.5–5 μm according to the supplier. Its hydroxyl, carboxyl and ether oxygens make graphite oxide hydrophilic and, thus, dispersible in water and compatible with the Boltorn hyperbranched polymers utilized. 

Hyperbranched polymer/GO nanohybrids composed of 50 wt% polymer and 50 wt% GO were prepared via a solution-intercalation method using deionized water (MilliQ, 18.2 MΩ·cm) as the solvent. The required amount of the polymer was first dissolved in water at room temperature, then the respective amount of the graphite oxide was added under continuous stirring in order to ensure that the GO layers were well dispersed. Following the mixing, the suspension was transferred in a petri dish and the solvent was evaporated in a vacuum oven until it was completely dry. The samples were thermally annealed at 120 °C overnight under vacuum to erase any metastable structures formed during solvent evaporation and to achieve equilibrium. The composition of the nanohybrids was chosen so that all galleries would be completely filled by the polymer, and there would be no bulk-like polymer chains outside of these completely filled galleries. In order to examine the effect of the in situ thermal reduction of GO on the structure and dynamics of the nanocomposites, the same nanohybrid samples were thermally annealed at ~200 °C overnight under vacuum.

### 2.2. Experimental Techniques

#### 2.2.1. X-ray Diffraction (XRD) 

The structural characterization of the pure polymers, the graphite oxide and the nanocomposites was performed by X-ray diffraction, using a RINT-2000 Rigaku Diffractometer. The X-rays are produced by a 12 kW rotating anode generator with a Cu anode equipped with a secondary pyrolytic graphite monochromator. The CuKα radiation was used with wavelength *λ_CuKα_* = 1.54 Å. All measurements were performed for diffraction angles 2θ from 1.5° to 30° with a step of 0.02°. Graphite oxide has a periodic structure; thus, characteristic (*00l*) diffraction peaks can be observed in its XRD pattern, related to the spacing of the layers according to Bragg’s law (*nλ*= 2*d_00l_*sinθ; *λ* is the wavelength of the radiation, *d_00l_* the interlayer distance, *n* the order of diffraction and 2θ the diffraction angle). In the case of the nanohybrids, the existence and the position of the respective peaks can provide information on the structure of the nanocomposite materials.

#### 2.2.2. Differential Scanning Calorimetry (DSC) 

The thermal properties of the nanocomposites and bulk polymers were investigated by a PL-DSC differential scanning calorimeter (Polymer Laboratories). The covered range of temperatures was between –80 °C and 150 °C for all materials and the heating/cooling rate was 10 °C/min in all cases. Two heating/cooling cycles were performed, and the transition temperatures were obtained from the second cycle to eliminate any thermal history effect. For the nanocomposites, an additional heating cycle up to 250 °C was performed to study the thermal reduction and de-oxygenation of GO. All measurements were performed in an inert atmosphere under nitrogen flow to prevent the decomposition of the samples. Controlled cooling was achieved using a liquid nitrogen cryostat. All DSC data in the present paper are shown in units of specific heat capacity, i.e., by dividing the measured heat flow by the heating rate and the appropriate mass.

#### 2.2.3. Thermogravimetric Analysis (TGA)

The thermal stability of the polymers and the effect of the nanoadditive on their decomposition behavior, as well as the GO reduction temperature in the presence of the polymers, was investigated by thermogravimetric analysis (TGA). An SDT600 TGA/DTA apparatus (TA Instruments) was utilized to detect the decomposition steps and determine the respective decomposition temperatures for the bulk polymers and for all nanohybrids. Heating scans were performed starting from room temperature to 600 °C at a heating rate of 10 °C/min under a flow of argon gas.

#### 2.2.4. Dielectric Relaxation Spectroscopy (DRS) 

Dielectric spectroscopy measures the complex dielectric permittivity ε*(ω) of a material as a function of angular frequency ω. A homogeneous electric field, oscillating at frequency ω, is applied to the sample, which is placed within the gap of a parallel plate capacitor. The molecular electric dipoles in the sample tend to be oriented by the field and, therefore, rotate according to their molecular mobility at the given frequency and temperature [72]. The complex dielectric permittivity:

(1) $$\varepsilon^{*}\left(\omega \right)=\varepsilon^{\prime }\left(\omega \right)-i\varepsilon^{\prime \prime }\left(\omega \right)$$

provides the storage (real part, $\varepsilon^{\prime }$) and the loss (imaginary part, $\varepsilon^{\prime \prime }$) dielectric contribution. A relaxation process is typically characterized by a peak in the spectrum of dielectric loss vs. frequency, centered at the relaxation frequency *f*_max_ = 1/(2πτ_max_), where τ_max_ is the most probable relaxation time of the process. The temperature dependence of the spectral features, such as relaxation time dielectric strength and peak width, provides information on the molecular dynamics of polar moieties, as the ones often found in polymer main chains or side groups, which can be influenced by both intra-and inter-molecular interactions as well as the properties and the interactions with the environment of the chain.

The dynamic measurements were performed utilizing a dielectric spectrometer (Alpha Dielectric Analyzer, Novocontrol GmbH, Montabaur, Germany), in the frequency range 10^−2^–10^7^ Hz. In the case of the pure polymers, the materials remained at 130 °C under vacuum for 24 h to eliminate humidity and at 100 °C for 30 min before the measurements and were thermally equilibrated at successively decreasing temperatures before the isothermal data collection. Fibers of fused silica of 100 μm in diameter were utilized as spacers. For the nanocomposites, the powder was pressed in pellets of 12 mm in diameter and 0.3–0.6 mm in thickness, and annealed at 140 °C in vacuum for 24 h. The pellets were placed between indium foils to optimize contact with the electrodes, and were kept at the highest starting temperature for 30 min before each measurement. Dielectric measurements were performed isothermally in the range of –110 to 110°C. The temperature was controlled via a heated flow of nitrogen gas from a Quatro Cryosystem (Novocontrol GmbH, Montabaur, Germany). During the measurements, the samples were constantly immersed in an inert nitrogen atmosphere. 

When multiple relaxation processes are present, the analysis of the measured dielectric spectra is performed utilizing a superposition of empirical Havriliak-Negami functions, HN, together with an additional ionic conductivity contribution at low-frequencies and high temperatures. The functional form of the fitting function is:

(2) $$\varepsilon^{*}\left(\omega \right)=\varepsilon_{\infty }+\underset{k}{\sum }\left[\frac{\Delta \varepsilon_{k}}{{\left\{1+{\left(i\omega \tau_{HN,k}\right)}^{\alpha_{k}}\right\}}^{\beta_{k}}}\right]+i\frac{\sigma_{dc}}{\varepsilon_{0}\omega }$$

where *k* is the index of the particular process, $\tau_{HN,k}$ its Havriliak-Negami relaxation time, $\Delta \varepsilon_{k}$ its dielectric (or relaxation) strength, $\varepsilon_{0}$ the dielectric constant of the vacuum, $\varepsilon_{\infty }$ the dielectric constant at frequencies much higher than the relaxation frequencies of all considered processes, and $\alpha_{k}$, $\beta_{k}$ (0 <$\;\alpha_{k}$*,* $\alpha_{k}\beta_{k}\;$≤ 1) are exponents describing the symmetric and asymmetric broadening of the distribution of relaxation times. It is noted that the Havriliak-Negami time, $\tau_{HN}$, and the position of the maximal loss, $\omega_{max}$ are related via the expression ωmax=1τHN[sinαπ2+2β]1α[sinαβπ2+2β]−1α [72]. $\sigma_{dc}$ is the static dc conductivity determined by the fitting of the dielectric spectra by means of Equation (2) or, equivalently, by the low frequency limit of the real part of the measured ac conductivity, σ’(ω).

## 3. Results

### 3.1. Static and Dynamic Behavior of Nanocomposites 

**Figure 1:**
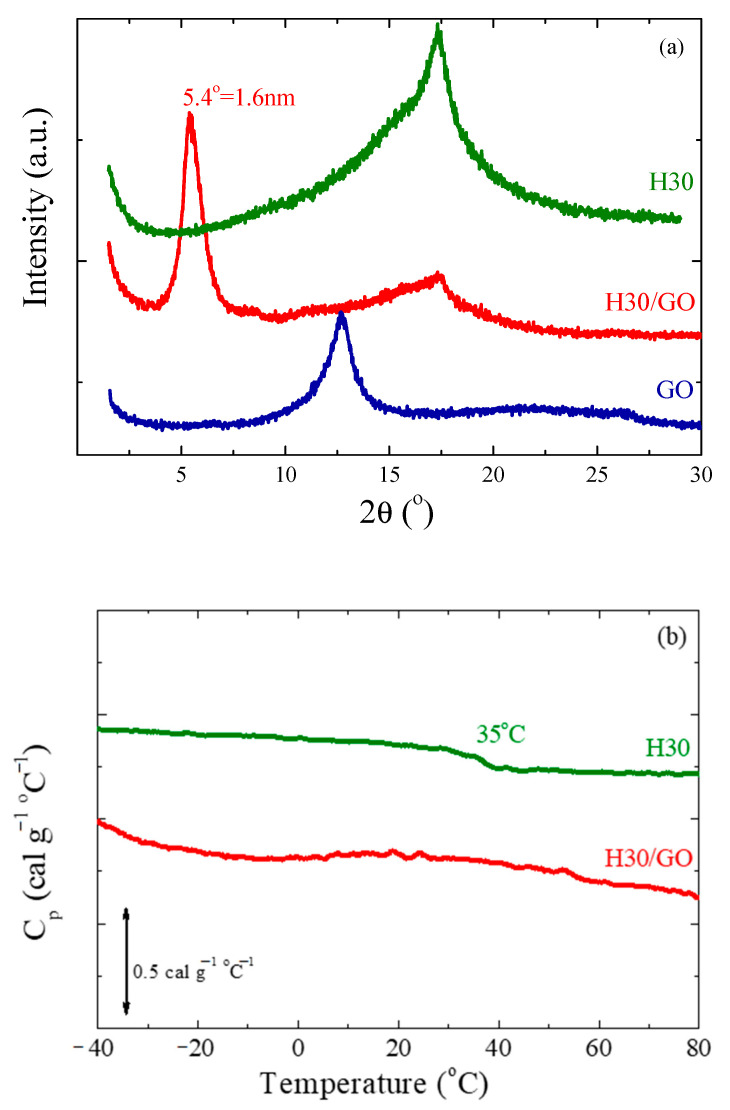
(**a**) X-ray diffractograms of 50 wt% H30/50 wt% graphite oxide (GO) together with neat H30 and neat GO. (**b**) Differential scanning calorimetry (DSC) thermograms of H30 and 50 wt% H30/50 wt% GO in terms of heat capacity. In both (**a**) and (**b**) the curves have been shifted vertically for clarity.

Figure 1a illustrates the structural characterization, obtained by X-ray diffraction, of the third generation of the hyperbranched polymers, Η30, as a representative of the three Boltorns of the graphite oxide (GO) and of the nanohybrid composed of 50 wt%H30 and 50 wt% GO. It is clear that the polymer is purely amorphous, showing only a broad diffraction peak; the sharper peak observed on top of the former at 2θ = 17.3 ± 0.1° that corresponds to a distance of ∼0.50 nm indicates a chain packing due to an extensive hydrogen bond network formed between hydroxyl hydrogens and hydroxyl oxygens (OH···OH) and hydroxyl hydrogens and carbonyl oxygens (OH···O = C) [63,64,73]. Similar diffraction curves are obtained for the other two polymers, H20 and H40 [28]. The XRD diffractogram of the GO confirms its layered structure, since it possesses a main diffraction peak at 12.7 ± 0.1° that corresponds to an interlayer distance of *d*_001_ = 0.70 nm. Application of the Scherrer equation to the diffractogram results in a coherence of the GO structure of ~10 layers per GO particle. An intercalated structure can be identified for the H30/GO nanocomposite, with 50 wt% H30 and 50 wt% GO, as evidenced by the shift of the main diffraction peak of GO towards lower angles, which is observed at *2*θ = 5.4 ± 0.1° corresponding to *d_001_* = 1.6 nm, i.e., there is an increase of the interlayer distance by ~0.9 nm. A diffraction peak at a similar angle is obtained for the H40 nanohybrid, whereas for the H20 nanocomposite, the peak is found at *2*θ = 6.4 ± 0.1°, corresponding to an interlayer distance of *d_001_* = 1.4 nm. The intercalated structures formed in the nanocomposites indicate favorable interactions between the polyester polyols and the surface of graphite oxide. This may explain the shorter interlayer distance for the second generation H20 polymer, since it possesses a smaller number of hydroxyl and ester groups, as can be seen in Scheme 1. It is noted that in the case of a polyester amide, with a lower number of functional groups and, thus, weaker interactions, mixed with GO in a similar composition, an intercalated structure was obtained, but with a significantly smaller interlayer distance [71].

The amorphous nature of the polymer is further confirmed by DSC measurements, shown in Figure 1b. The measurement was performed following thermal annealing of the polymer at 120 °C. The curve of specific heat capacity, c_p_, exhibits a single step that corresponds to the glass transition temperature at T_g,H30_ = 35 ± 2 °C, and no indication of melting; the error is associated with the breadth of the transition and the remaining residual humidity in the sample. The thermograms are very similar for the other two polymers, with the glass transition temperature being a function of the generation at T_g,H20_ = 14 ± 2 °C for H20 [28] and T_g,H40_ = 46 ± 2°C for H40; this may be attributed to the different molecular weight and the different degree of branching. On the other hand, the step in the specific heat capacity at the transition seems to be constant for the three generations having the value of ∆c_p_ = 0.11cal.gr^−1^ °C^−1^. Figure 1b shows the DSC thermogram of the nanohybrid composed of 50 wt% H30 and 50 wt% GO, as well. To calculate the heat capacity in the case of the nanocomposites, only the mass of the polymer was taken into account. The H30/GO nanocomposite, as well as the nanohybrids with all three Boltorn polymers, exhibit no identifiable glass transition, indicating that at this polymer concentration, all or most of the polymer chains are confined and/or reside in the proximity of the GO surface, inhibiting the glass transition.

**Figure 2:**
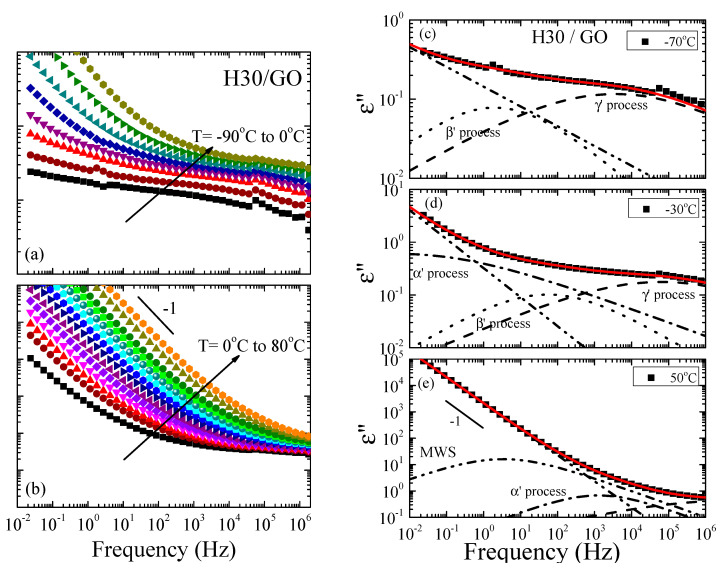
(**a**,**b**) Frequency dependence of the imaginary part of the complex permittivity, ε”, for 50 wt% H30/50 wt% GO nanohybrid in the temperature range of −90 °C to 0 °C (**a**) and 0 °C to 80 °C (**b**). (**c**–**e**) Analysis of the imaginary part of the dielectric permittivity, ε”, for the H30/GO with 50 wt% polymer at −70 °C (**c**), −30 °C (**d**) and 50 °C (**e**). The processes needed for the deconvolution of the spectra are shown with dashed (γ’), dotted (β’), dash-dotted (α’) and dash-dot-dot (conductivity) lines, whereas the solid lines are the summation of the processes (best fit).

Figure 2a,b show the frequency dependence of the imaginary part of the complex permittivity, ε”, for the H30/GO nanocomposite as a function of frequency over a wide range of temperatures both below (Figure 2a) and above (Figure 2b) the glass transition temperature. The respective measurement for the H30 polymer is shown in Figure S1 in the Supplementary Material. At low temperatures, the spectra exhibit multiple relaxation processes, as evidenced by the breadth of the curves, similar to the data for the pure H30 polymer; nevertheless, the curves have a very different shape and lower dielectric strength. In this case, as well, the processes may be attributed to motions of the functional groups present in the molecules, such as the hydroxyl rotation and the ester reorientation. At higher temperatures, but still below the glass transition temperature of the neat polymers (no calorimetric T_g_ has been observed by DSC for the nanohybrids), an additional relaxation process appears in the spectra. At even higher temperatures, another process appears, together with a conductivity contribution, in all nanocomposites, that may be attributed to the Maxwell-Wagner-Sillars (MWS) polarization effect, due to the presence of surfaces; the latter appears commonly in dielectric spectra of inhomogeneous materials like suspensions or colloids, biological materials, phase separated polymers, blends, and crystalline or liquid crystalline polymers [72].

Figure 2c–e demonstrate the representative analysis of the spectra of H30/GO for three temperatures: two below −70 °C (Figure 2c) and −30 °C (Figure 2d), and one above 50 °C (Figure 2e) the T_g_ of the neat H30 polymer that was performed utilizing a superposition of Havriliak–Negami functions as described in the experimental techniques section. The respective analysis for the neat H30 at the same temperatures is shown in Figure S2 of the Supplementary Material. From the analysis, it appears that multiple relaxation processes are necessary to obtain a good fit to the data in the case of the nanocomposites, similarly, to the case of the polymers and all relaxation processes identified for the polymers should be included in the analysis for all nanocomposites, as well. Moreover, the behavior is qualitatively very similar between the three nanohybrids. To differentiate the processes of the nanocomposites from those in the bulk, they were named by the same Greek letters followed by a prime apex (α’, β’, γ’). From the spectra at low temperatures, the shape and relaxation strength parameters are determined for the γ’- and β’-processes of the nanocomposites. For all three generations, the β exponent of all processes in the nanocomposites was fixed at 1.0. For the γ’ process, the α exponent changes with temperature in the range 0.23−0.3 as does the dielectric strength in the range 0.7−2.7 (from −90 to 20 °C); the dependencies were derived from the low temperature spectra. For the β’ process, the α exponent takes values around 0.41–0.45 whereas the dielectric strength, Δε~0.13–0.60, is quite low for all the three generations. At higher temperatures, but still below the bulk polymer glass transition temperature, the intermediate α’ process appears (dash-dotted line) and, at even higher temperatures, the slower MWS process becomes evident. For both the intermediate and the slow processes, the β exponent was fixed at 1.0. The α exponent of the α’ process for all three nanocomposites is around 0.26−0.56 and Δε decreases from 5 to 1.2; for the slow process, the value of the shape exponent α is around 0.5, whereas ∆ε increases from 70–80 for the H30 nanocomposite, while it increases from 30 to 37 for the H20 and H40. At low frequencies and high temperatures, the addition of a contribution due to ionic conductivity is necessary with a ω^-n^ dependence with the exponent n~0.95−0.98.

**Figure 3:**
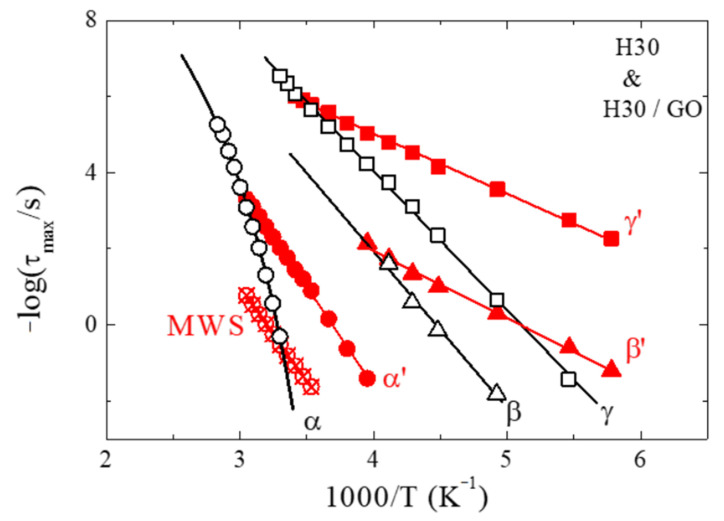
Arrhenius relaxation map for the H30 hyperbranched polymer and the H30/GO nanocomposite with 50 wt% polymer. The fast and slow sub-*T*_g_ process are shown as squares and triangles whereas the α-relaxation is shown as circles. All relaxation times for H30 are represented with black open symbols, and the respective ones of the nanohybrid as red solid symbols. The lines correspond to the fitting of the data with a Vogel-Fulcher-Tammann (VFT) equation for the bulk α-relaxation and with an Arrhenius equation for the remaining processes. The errors in the times are smaller than the size of the points.

Figure 3 shows the relaxation times of H30 and H30/GO nanohybrid that resulted from the analysis discussed above in an Arrhenius representation. For the neat H30 hyperbranched polymer, the two sub-*T*_g_ relaxation processes can be identified as the γ- and the β-processes, whose relaxation times follow Arrhenius temperature dependencies, $\tau =\tau_{0}exp\left[E_{a}/RT\right]$. The γ-process is attributed to local fluctuations and to the rotation of the hydroxyl groups, whereas the β-process, which is seen at fewer temperatures, to the ester reorientation. Both processes have qualitatively similar temperature dependencies of their relaxation times: the activation energy of the faster one is E_γ,H30_ = 69.5 ± 1.0 kJ/mol, whereas that of the slower one is E_β,H30_ = 81 ± 9 kJ/mol. It is noted that, if we consider all three polymers, there is no dependence of the activation energy on the generation (E_γ,H20_ = 65.0 ± 1.5 kJ/mol [28], and E_γ,H40_ = 66.5 ± 1.5 kJ/mol), which means that the motion of the hydroxyl groups is not affected by the local density of the polymer due to the small size of these functional groups. These values are lower than what has been reported in the literature for similar motions [65,67,74]; nevertheless, they are higher in comparison to the respective local processes observed for linear polymers [25]. On the contrary, the activation energies for the β-process show some dependence on the generation (E_β,H20_ = 70 ± 3 kJ/mol [28] and E_β,H40_ = 86 ± 2 kJ/mol), indicating that the reorientation of the carbonyl groups becomes more difficult as the material becomes denser. The large values of these activation energies can be understood to be due to the existence of functional groups that can form both intra- and inter-molecular hydrogen bonds, which hinder their motion. At higher temperatures and above the glass transition temperature of H30, the segmental process appears, as expected. Its temperature dependence follows the Vogel-Fulcher-Tammann (VFT) equation, $\tau =\tau_{0}exp\left[B/\left(T-T_{0}\right)\right]$, and the resulting parameters are τ_0_ = 1 × 10^−13^ s (kept fixed to minimize error), B = 2113 ± 24 K, and T_0_ = 233.5 ± 1.0 K. This process appears in the data of H20 [28] and H40 as well; nevertheless, its relaxation times reflect the difference of the calorimetric *T*_g_’s among the three generations. Moreover, the Vogel temperature, T_0_, that results from the VFT fits, increases with the generation, showing a similar dependence with that of the calorimetric glass transition temperature, as anticipated; furthermore, the fragility parameter D = B/T_0_ results to 8.6, 9.0, and 9.4 for H20, H30, and H40, respectively, denoting that the increase in the generation results in slightly stronger glasses [28].

For the H30/GO nanocomposite, the two sub-*T*_g_ processes show a similar behavior and follow Arrhenius temperature dependencies like the neat polymer. Nevertheless, their relaxation times are faster and their activation energies are much lower than the respective ones for the neat polymer, namely, E_γ’,H30_ = 31 ± 1.0 kJ/mol and E_β’,H30_ = 35 ± 2 kJ/mol. These two processes are attributed to the motion of the polar hydroxyl and the reorientation of the carbonyl groups of the polymer chains that are confined between the layers of graphite oxide. The faster relaxation times and the lower activation energies can be understood to be due to the decreased ability of the molecules to form hydrogen bonds, since the appropriate angles and distances are not easily accommodated within the less than 1 nm interlayer distance. It is noted that these values for the activation energies are usually obtained for the sub-*T*_g_ processes of non-hydrogen bond forming polymers. Thus, both the hydroxyl motions and the carbonyl reorientation become easier in confinement. Moreover, similar to the neat polymers, the relaxation times and the activation energies are very similar for all nanocomposites; the latter obtain the values E_γ’,H20_ = 36 ± 1.5 kJ/mol and E_γ’,H40_ = 30 ± 1.0 kJ/mol for the γ’-process and E_β’,H20_ = 33.5 ± 2.0 kJ/mol and E_β’,H40_ = 28 ± 2 kJ/mol for the β’-process. At higher temperatures, the relaxation process that would correspond to the segmental relaxation of H30 appears below the glass transition temperature of the neat H30 (note that no *T*_g_ has been observed in the DSC measurement of H30/GO) and its relaxation times show an Arrhenius temperature dependence (with an activation energy of E_α’,H30_ = 98 ± 3.0 kJ/mol) instead of the usual VFT dependence. The dielectric strength of this process, however, decreases with increasing temperature, as is typical for the segmental relaxation. The behavior is similar for the other two confined polymers, with the respective activation energies E_α’,H20_ = 99.5 ± 5.0 kJ/mol and E_α’,H40_ = 97 ± 2.0 kJ/mol, i.e., very similar for the three generations. Moreover, the relaxation times are quite similar between the generations, although a slight trend is evident with somehow slower segmental dynamics with increasing generation; nevertheless, this dependence is much weaker than the one that would be anticipated if it were due to the different *T*_g_’s of the neat polymers. Due to the different temperature dependence of the segmental process in the nanocomposite, the α’-process tends to become slower than the α-process of the bulk polymer at high temperatures. This crossing is more clearly observed for the H20/GO nanocomposite, because the dependence of the relaxation time of the α’ relaxation on generation (in the same temperature range) is weaker than the difference between the glass transition temperatures and, thus, of the T_0_′s of the bulk polymers. Finally, at even higher temperatures, the slow MWS process appears, in the nanocomposites, due to interfacial polarization because of the large number of interfaces that exist in the nanocomposites and to ion species trapped in their proximity. 

The dynamic behavior of the three generations of the Boltorn hyperbranched polymers was investigated in confinement, when the polymers were mixed with a different layered material and, more specifically, layered sodium montmorillonite (Na^+^-MMT) [28]. Intercalated nanocomposites were obtained in that case, as well. The dynamic response of the confined polymers results is very similar in the two systems; two sub-*T*_g_ processes are observed, that become faster with lower activation energy under confinement, whereas the segmental process appears at temperatures lower than the glass transition temperatures of the neat polymers and shows an Arrhenius, rather than a VFT, temperature dependence. One difference between the two systems is that the generation dependence looks stronger for the nanocomposites with Na^+^-MMT than for those with the GO. On the contrary, in a separate work, a polyester amide hyperbranched polymer, named Hybrane, was confined between the layers of Na^+^-MMT and GO, and a different behavior was obtained for the two layered systems, because of the different interfacial interactions [71]. Hybrane possesses a different number, as well as different kinds, of functional groups, and therefore a different ability of hydrogen bond formation, with respect to Boltorn. The two sub-*T*_g_ processes in the case of GO exhibited activation energies similar to the bulk polymers, whereas they resembled the one obtained with the Boltorns in the case of Na^+^-MMT. An intermediate process related to the branch movement was observed (never observed for the Boltorns) for the neat Hybrane and the Na^+^-MMT nanohybrid, whereas the segmental process under confinement was faster for the nanocomposites with GO and slower for those with Na^+^-MMT than the segmental motion of the polymer; nevertheless, in both cases, its temperature dependence in confinement was of the Arrhenius type. 

### 3.2. Static and Dynamic Behavior of Nanocomposites after Thermal Reduction 

**Figure 4:**
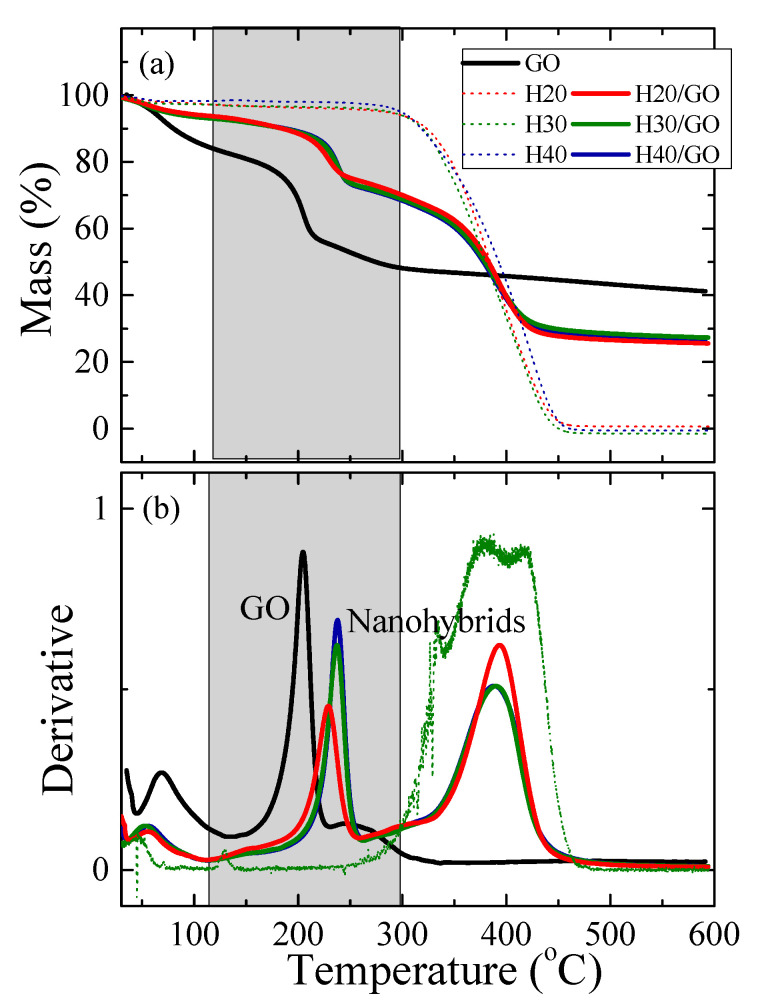
(**a**) Temperature dependence of the mass loss as measured by thermogravimetric analysis (TGA) for the neat polymers, the neat GO and the nanocomposites with the three hyperbranched polymers containing 50 wt% GO. (**b**) The respective derivatives of the mass loss curves for the GO and the nanocomposites; for the polymers the derivative of only H30 is shown for clarity.

Following the investigation of the static and dynamic behavior of the hyperbranched polymers when confined between the layers of graphite oxide, an attempt was made to change the structure of the nanoadditive in the presence of the polymer, and study how this would affect both the structure of the nanohybrid as well as the dynamic response of the polymer. It is well known that thermal annealing of GO at temperatures above 190 °C results in its de-oxygenation, i.e., the consecutive removal of the various oxygen species from its layers. At the same time, thermal reduction in the presence of the polymer would reveal the influence of the polymer on the reduction mechanism of the GO. Figure 4 shows the thermogravimetric analysis measurements in terms of mass loss as a function of temperature for the three polymers of the different generations, for the GO and for the nanocomposites with composition 50 wt% polymer and 50 wt% GO. The three polymers are completely stable up to ~280 °C; at higher temperatures, their decomposition starts, proceeding in multiple steps. Initially, there is a small decomposition step with a most probable decomposition temperature that shows a strong dependence on the generation being *T*_d_~348 ± 2 °C for H20, *T*_d_~333 ± 1 °C for H30 and *T*_d_~320 ± 2 °C for H40, i.e., it decreases with increasing the generation of the hyperbranched polymer, whereas the corresponding mass loss increases with generation. At higher temperatures there are two additional decomposition steps, at ~380 ± 2 °C and ~420 ± 2 °C. The former has an amplitude decreasing with the generation, whereas the opposite trend is observed for the latter. All decomposition temperatures were identified utilizing the first derivative of the corresponding mass loss curves (shown in Figure 4b as well, for H30).

As far as the GO and the nanohybrids are concerned, an initial loss of weight is observed (~15% of its total weight for the GO and ~6% for the nanocomposites) for temperatures up to 100 °C due to the removal of water molecules adsorbed on the materials due to their hydrophilic character. Above 150 °C, neat GO shows a double decomposition/de-oxygenation step due to the different types of oxygen species (such as hydroxyl, carbonyl and ether) present on the GO surface; the first corresponds to ~25% of its total weight and occurs at 205 ± 2 °C whereas the latter corresponds to ~10% of the weight and is observed at 255 ± 2 °C. The de-oxygenation temperatures are derived from the most probable decomposition temperatures obtained by the derivatives of the mass loss curves (Figure 4b). Above 600 °C, there is a ~41wt% solid remaining. For the nanohybrids, Figure 4 shows that the thermal behavior of the nanocomposites with the three generations of Boltorn is very similar, with small or no observable dependence of the decomposition temperatures on generation. The first decomposition step occurs at 228 ± 2 °C for H20/GO and at 238 ± 2 °C for H30/GO and H40/GO and corresponds to the reduction of GO in the presence of the different polymers; this takes place at a considerably higher temperature than the respective one of the neat GO. On the other hand, the second clear decomposition step present in the hybrids that corresponds to the decomposition of the polymer, is found at ~390 ± 2 °C (for all three generations), which is significantly lower than that of the neat polymers. This indicates that the oxygen groups that are removed from the GO surface react with the functional groups of the polymers in such a way as to favor their decomposition. Above 450 °C, a solid residue of ~26% is obtained, which is accounted for by the composition of the nanohybrids.

**Figure 5:**
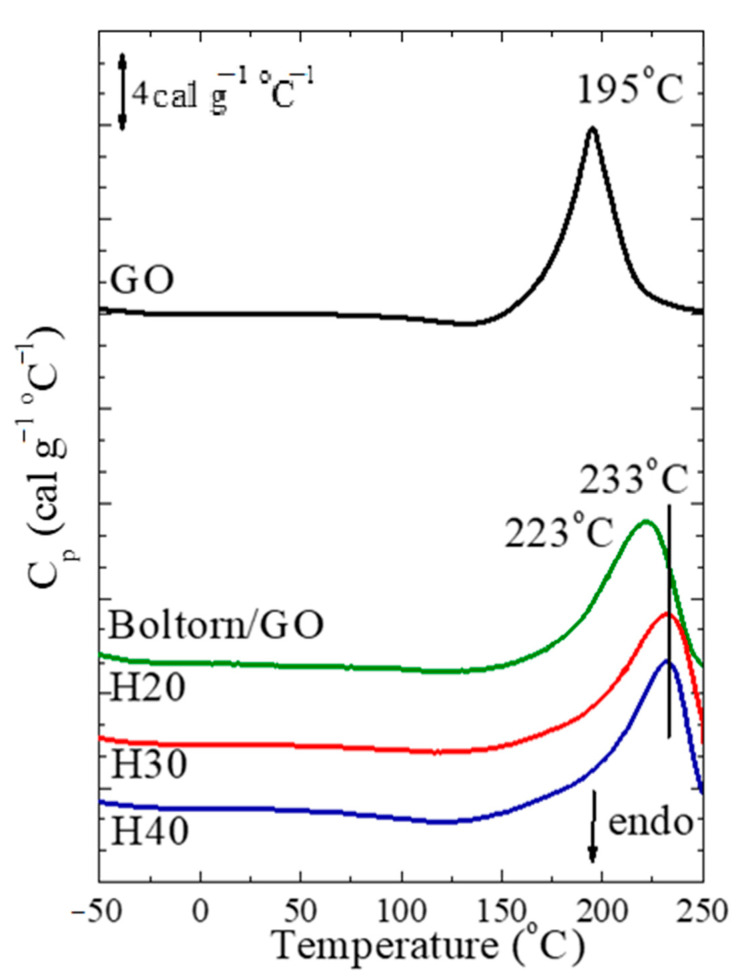
DSC thermograms shown in units of specific heat capacity measured during heating for the neat GO and the nanocomposites with the three hyperbranched polymers. The curves have been shifted vertically for clarity. It is noted, that in this case the mass utilized for the calculation of c_p_ is the mass of graphite oxide.

To verify these results, DSC measurements were performed to record the exothermic reduction process for the GO and the three nanocomposites and the thermograms, are shown in Figure 5. The neat GO does not show any thermal transition up to 140 °C; at that temperature an exothermic transition begins with a peak at 195 ± 1 °C. For the nanohybrids, such an exothermic transition is observed at much higher temperatures, in accordance with the TGA measurements; it occurs at 223 ± 2 °C for H20/GO and at 233 ± 2 °C for H30/GO and H40/GO. The measurements indicate that increasing the generation, and subsequently, the number of hydroxyl and ester groups, results in a significant increase in the de-oxygenation temperature. However, in most of the studies published in the past, an opposite result was found, especially when strong favorable interactions between the polymer and the GO surfaces exist [75] and/or in the presence of polar or aromatic groups in the polymers [56]. Moreover, in a previous work from our group [71], in which the hyperbranched polyester amide Hybrane was utilized to synthesize nanocomposites with GO, the de-oxygenation temperature in the presence of the polymer was found to be ~15°C lower than that of the GO. The more favorable interactions of the polyester amide with the graphite oxide layers were evident from the slower segmental dynamics observed under confinement in that case, when compared to the respective one of the neat polymer, in contrast to the faster segmental dynamics that are recorded for H30/GO in the present work when compared to H30, as discussed in Figure 5. Consequently, it is the presence, as well as the kind, of functional groups comprising the polymer, that affects the reduction temperature and defines not only the shift of the critical temperature, but its direction as well.

**Figure 6:**
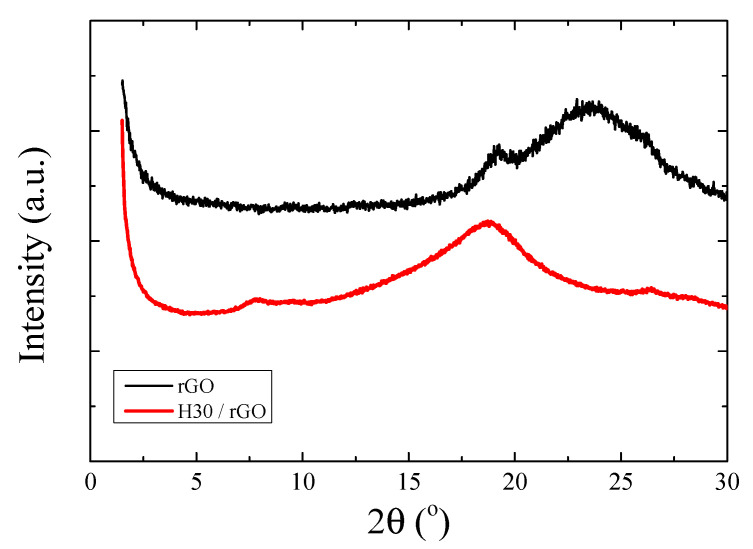
X-ray diffractograms of reduced graphite oxide (rGO) and H30/rGO after thermal reduction at 215 °C. The curves have been shifted vertically for clarity.

Thermal reduction and, thus, de-oxygenation of GO in the presence of the Boltorn hyperbranched polymers occurs at ~220–230 °C. Removal of the oxygen groups is anticipated to reduce the hydrophilicity of the nanoadditive and, therefore, alter its interactions with the hydrophilic polymers. Figure 6 shows X-ray diffraction measurements of the reduced graphite oxide (rGO) and of the nanocomposites following thermal reduction at 215 °C for 12 h; note that the latter was not attempted at higher temperatures to preserve the thermal stability of the polymers. It is clear that both the neat rGO and the nanohybrid composed of 50 wt% H30 and 50 wt% GO, after reduction of the GO, have lost their layered structure, as shown in Figure 2a, since only a broad halo in their diffraction curve is observed, reflecting the dispersion of reduced GO layers within the amorphous polymer matrix. The very broad peak of rGO is found at 2θ = 23.5°, whereas a small peak at 2θ = 19.2° is also evident, indicating the presence of oxygen-containing moieties or water molecules. The amorphous halo of the nanohybrid is found at 2θ = 18.7°, which is intermediate between the ones for the amorphous H30 and the rGO.

**Figure 7:**
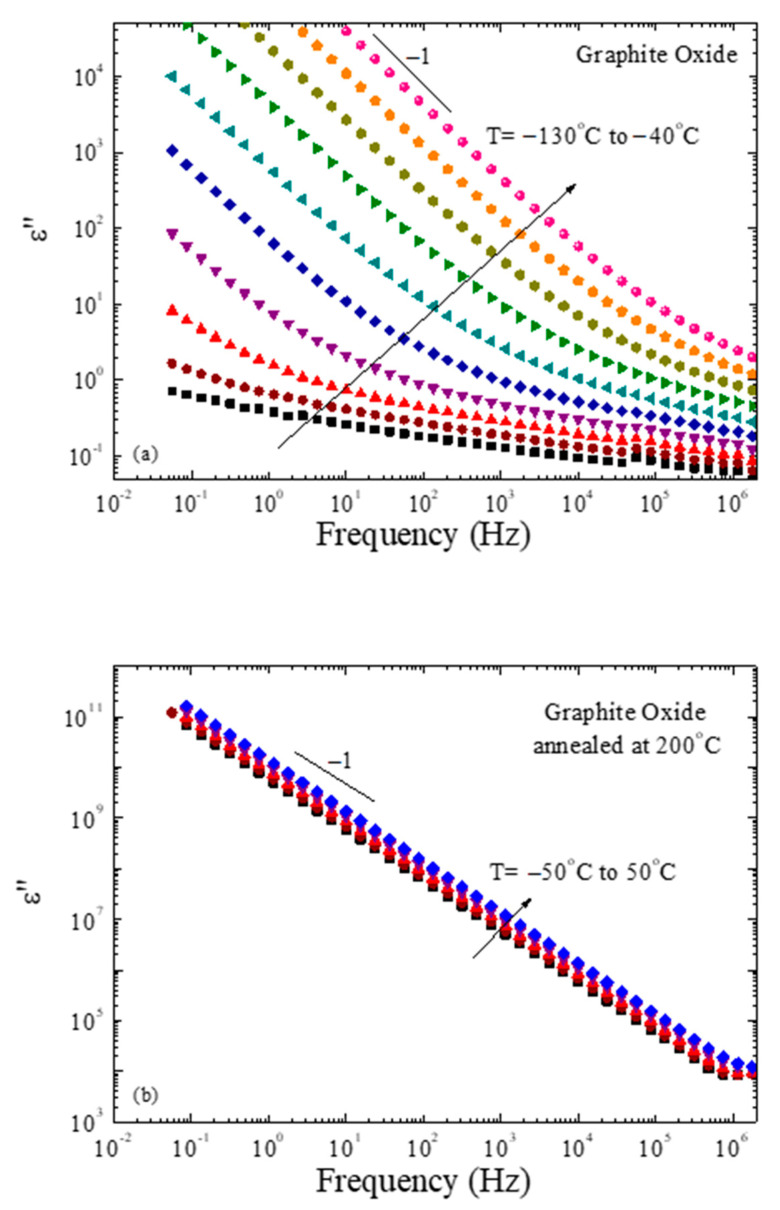
Frequency dependence of the imaginary part of the complex permittivity, ε”, of GO (**a**) prior to and (**b**) after thermal annealing.

Graphite oxide is considered an insulator due to defects in the parent graphene layers that are caused by the oxidation reactions; however, thermal reduction apparently creates a percolated network of rGO layers, which is anticipated to increase its conductivity. Dielectric spectroscopy was utilized to measure the dielectric response of graphite oxide before and after thermal reduction. Figure 7a shows the imaginary part of the dielectric permittivity of GO that was kept in vacuum overnight to remove any traces of humidity. A relaxation process of very low dielectric strength is evidenced only at the lowest temperatures. This can be associated with the motion of the functional groups present on the GO surface. It should be mentioned that the relaxation times resulting from the analysis of these spectra are much faster than any relaxation process obtained for either the polymers or the nanocomposites discussed in the previous section, and do not interfere in any way with the results presented there. As the temperature increases, the larger part of the spectra is covered by the contribution of conductivity, shown by the ω^−1^ dependence of the ε” curve. Dielectric measurements were performed for GO following thermal annealing at 200 °C (and, thus, after its reduction). The imaginary part of its dielectric permittivity is shown in Figure 7b as a function of temperature. It is clear that the de-oxygenation and the change of the GO structure results in a completely different dielectric response, since the graphene oxide layers after reduction are conductive and, thus, the whole spectra at all temperatures, including room temperature, are dominated by the conductivity over the whole frequency regime.

**Figure 8:**
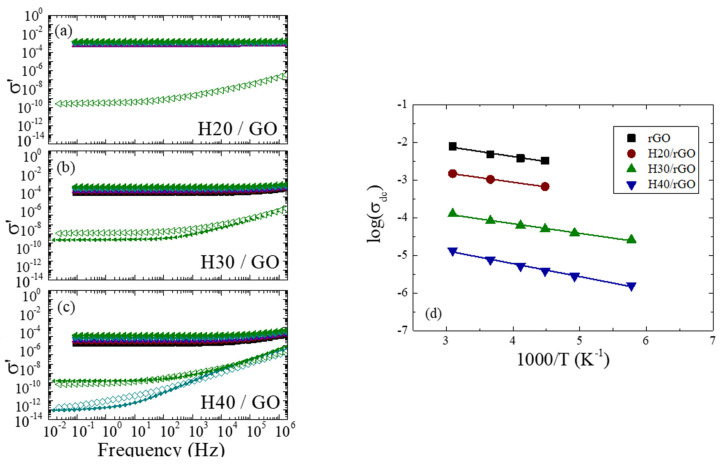
(**a**–**c**) Frequency dependence of the real part of the complex ac conductivity for H20/GO (**a**), H30/GO (**b**) and H40/GO (**c**) for different temperatures. (■) −100 °C, (●) −70 °C, (▲) −50 °C, (▼) −30 °C, (♦) 0 °C and (◄) 50 °C. Solid symbols are the measurements after the thermal annealing (i.e., after reduction of GO) whereas open symbols are the respective measurements at the same temperatures before the thermal annealing. The line-plus-symbol curves are measurements for the neat polymers. (**d**) Temperature dependence of the dc conductivity for GO, H20/GO, H30/GO and H40/GO. It is noted that the errors in the values of the conductivities σ_dc_ are smaller than the size of the points.

After thermal reduction, carbon atoms of graphene oxide recover their sp^2^ hybridization, and become electrically conductive. It is interesting to examine how thermal reduction affects the conductivity of the nanocomposites as well, which based on the X-ray diffraction measurements shown in Figure 6, comprise of an exfoliated network of reduced graphene oxide layers. Figure 8a−c show the frequency dependence of the real part of the complex ac conductivity for the three nanocomposites σ’(ω) over a broad temperature range, from −100 to 50 °C. The respective measurements at specific temperatures of nanocomposites prior to the reduction, as well as of the three neat polymers, are included for comparison. It is clear that, for all three systems, conductivity is significantly increased by orders of magnitude, following the reduction of GO. Additionally, a dependence of the conductivity on the generation is observed, since its values decrease when the generation number increases. At the same time, a temperature dependence of the conductivity is observed, without a significant effect on the generation. Thus, it can be inferred that the sheets of the additive can provide percolated pathways for electron transfer, making the nanocomposites electrically conductive.

Figure 8d shows a comparison of the dc conductivity for the three nanocomposites, in an Arrhenius representation, to that of the rGO. The dc conductivities for rGO are determined by the analysis of the dielectric spectra of Figure 7b utilizing Equation (2) (although the data are dominated by the conductivity contribution) whereas the dc conductivities of the nanocomposites by the low frequency limit of the σ’(ω) data of Figure 8a−c. The temperature dependence of the dc conductivity, when conduction is due to some carrier hopping mechanism, as usual in disordered systems, is given by Mott’s law $\sigma_{dc}=\sigma_{0}e^{-{\left(\frac{T^{*}}{T}\right)}^{\gamma }}$[76], where T^*^ is a characteristic temperature related to the localization and density of the states contributing to conduction and is essentially related to the state of disorder in the system and the exponent γ is related to the dimensions and the variability of the transport process; γ = 1 for a fixed range hopping model whereas γ < 1 for a variable range one with the values γ = 1/2, 1/3 and 1/4, indicating conduction in 1, 2 or 3 dimensions, respectively [77]. The data of Figure 8d show a linear dependence of $log\sigma_{dc}$ with 1/T, signifying that γ = 1; however, the narrow temperature range of the measurements should be taken into consideration. In a similar study, nanocomposites based on polyvinylidene fluoride (PVDF) with different carbon-based additives, at different concentrations, were similarly characterized by dielectric spectroscopy [78] and the data were consistent with the formation of a percolated network.

Therefore, the dc conductivity data for the rGO and the nanohybrids show an Arrhenius temperature dependence (over the specific temperature range) with the values for the rGO being the highest ones, as expected. A significant dependence of the dc conductivity is observed on the generation of the hyperbranched polymers with the conductivity increasing with decreasing polymer size and number of functional groups and, thus, strength of the polymer/surface interactions, which indicates that the percolated network of the rGO sheets created depends on these parameters. It should be noted that ionic conduction is, in principle, expected in our polymers whereas electronic conductivity within rGO is anticipated. The typical ω^−1^ frequency dependence of the ε” (ω) data at high temperatures and low frequencies, together with the presence of MWS interfacial polarization in the polymer/GO nanocomposites, support the presence of ionic conduction. However, the percolated networks of conductive rGO significantly increase the sample conductivity, adding electronic conduction by a hopping process taking place across the conductive platelets. Furthermore, the data show very similar temperature dependencies for all systems, which is expressed with very similar values of the characteristics T^*^ or, alternatively, with very similar activation energies; the latter indicates a similar degree of disorder in these systems. The values of the activation energies for rGO, H20/rGO and H30/rGO are 2.2, 2.1 and 2.1 kJ/mol, respectively, and it is only for the nanohybrid H40/rGO with the larger polymer size that it becomes somewhat larger (2.9 kJ/mol), indicating an increased difficulty in the conduction mechanism through the percolated rGO network.

## 4. Conclusions

The static and dynamic behavior of nanohybrids composed of three different generations of Boltorn hyperbranched polymers and graphite oxide, before and after thermal annealing, was investigated. The nanohybrids with GO possess intercalated structure because of the favorable interactions between the polymers and the solid surfaces, due to their functional groups which renders both materials hydrophilic. The thickness of the intercalated polymer film was found constant at ~0.9 nm for the two larger polymers, whereas it was somewhat smaller (0.7 nm) for the polymer with the smallest number of functional groups, and thus with the weaker interactions with the GO surface. The glass transition temperature for the intercalated chains was completely suppressed in all cases. Thermal reduction of GO in the presence of the polymers resulted in an increase of the GO de-oxygenation temperature by more than 25 °C, but in a similar decrease of the polymer decomposition temperature as well. The reduction resulted in a modification of the polymer/surface interactions, and in the alteration of the nanohybrid structure, which loses its periodicity and leads to reduced graphite oxide layers dispersed within the polymer matrix. The dynamics of the intercalated polymer chains before thermal annealing showed the same relaxation processes as the ones observed in the neat polymers, but, nevertheless, with different characteristics. Both sub-*T*_g_ processes, identified as the hydroxyl motion and the ester reorientation, are found with significantly lower activation energies in comparison to the respective motions of the bulk polymers, due to the smaller number of intra- and inter-molecular hydrogen bonds that can be formed because of the severe confinement within the GO galleries. The segmental dynamics of the confined chains show an Arrhenius temperature dependence and a weak dependence on the generation of the hyperbranched polymers, which differs from both the VFT dependence and the generation dependence obtained for the neat polymers, which reflected the difference in their glass transition temperatures. Following thermal reduction of the GO, a significant increase of the conductivity by four to six orders of magnitude is observed, with respect to either the neat polymers or the intercalated nanocomposites, due to the percolated structure of the rGO flakes formed within the polymer matrix. Conductivity shows a significant dependence on the generation of the hyperbranched polymers, being attenuated in the case of the largest molecules. It is emphasized that dispersing graphene (or reduced graphene oxide) within a not very hydrophobic polymer is not possible; thus, we illustrate here a way to disperse the graphene oxide (GO) in a hydrophilic polymer, reduce the GO in the presence of the polymer and get a hybrid with a percolated network of reduced GO with a greatly increased conductivity. It should be noted, however, that this results further than the increase of the conductivity to a decrease of the polymer decomposition temperature as well; nevertheless, this temperature remains close to 400 °C, so the decrease is not anticipated to affect its utilization.

## Data Availability

The data included in this paper are available upon request to the corresponding author.

## Supplementary Materials

The following are available online at https://www.mdpi.com/2073-4360/13/7/1008/s1, Figure S1: Imaginary part of the complex permittivity, ε”, as a function of frequency, ω, for H30 in the temperature range of −50 °C to 0 °C (a), 0 °C to 60 °C (b): Figure S2: Imaginary part of the dielectric permittivity, ε", for the H30 at −70 °C (a), −30 °C (b) and 50 °C (c). The processes needed for the deconvolution of the spectra are shown with dashed (γ), dotted (β), dash-dotted (α) and dash-dot-dot (conductivity) lines, whereas the solid lines are the summation of the processes (best fit).

## References

[1] Schmidt S., Shah D., Giannelis E.P. (2002). New advances in polymer/layered silicate nanocomposites. Curr. Opin. Sol. Stat. Mater. Sci..

[2] Moniruzzaman M., Winey K.I. (2006). Polymer nanocomposites containing carbon nanotubes. Macromolecules.

[3] Akcora P., Liu H., Kumar S.K., Moll J., Li Y., Benicewicz B.C., Schadler L.S., Acehan D., Panagiotopoulos A.Z., Pryamitsyn V. (2009). Anisotropic self-assembly of spherical polymer-grafted nanoparticles. Nat. Mater..

[4] Jancar J., Douglas J.F., Starr F.W., Kumar S.K., Cassagnau P., Lesser A.J., Sternstein S.S., Buehler M.J. (2010). Current issues in research on structure—Property relationships in polymer nanocomposites. Polymer.

[5] Mittal V. (2014). Functional polymer nanocomposites with graphene: A review. Macrom. Mater. Eng..

[6] Kinloch I.A., Suhr J., Lou J., Young R.J., Ajayan P.M. (2018). Composites with carbon nanotubes and graphene: An outlook. Science.

[7] Ethier J.G., Drummy L.F., Vaia R.A., Hall L.M. (2019). Uniaxial Deformation and Crazing in Glassy Polymer-Grafted Nanoparticle Ultrathin Films. ACS Nano.

[8] Koh C., Grest G.S., Kumar S.K. (2020). Assembly of Polymer-Grafted Nanoparticles in Polymer Matrices. ACS Nano.

[9] Hegde M., Yang L., Vita F., Fox R.J., van de Watering R., Norder B., Lafont U., Francescangeli O., Madsen L.A., Picken S.J. (2020). Strong graphene oxide nanocomposites from aqueous hybrid liquid crystals. Nat. Commun..

[10] Ganguly S., Bhawal P., Ravindren R., Das N.C. (2018). Polymer Nanocomposites for Electromagnetic Interference Shielding: A Review. J. Nanosci. Nanotechnol..

[11] Pastoriza-Santos I., Kinnear C., Perez-Juste J., Mulvaney P., Liz-Marzan L.M. (2018). Plasmonic Polymer Nanocomposites. Nat. Rev. Mater..

[12] Feldman D. (2018). Polymer nanocomposites for tissue engineering, antimicrobials and drug delivery. Biointerface Res. Appl. Chem..

[13] Abbas M., Buntinx M., Deferme W., Peeters R. (2019). (Bio)polymer/ZnO Nanocomposites for Packaging Applications; A Review of Gas Barrier and Mechanical Properties. Nanomaterials.

[14] Melinte V., Stroea L., Chibac-Scutaru A.L. (2019). Polymer Nanocomposites for Photocatalytic Applications. Catalysts.

[15] Giliopoulos D., Zamboulis A., Giannakoudakis D., Bikiaris D., Triantafyllidis K. (2020). Polymer/Metal Organic Framework (MOF) Nanocomposites for Biomedical Applications. Molecules.

[16] Feldman D. (2021). Polyurethane and Polyurethane Nanocomposites: Recent Contributions to Medicine. Biointerface Res. Appl. Chem..

[17] Chrissopoulou K., Andrikopoulos K.S., Fotiadou S., Bollas S., Karageorgaki C., Christofilos D., Voyiatzis G.A., Anastasiadis S.H. (2011). Crystallinity and Chain Conformation in PEO/Layered Silicate Nanocomposites. Macromolecules.

[18] Carr J.M., Langhe D.S., Ponting M.T., Hiltner A., Baer E. (2012). Confined crystallization in polymer nanolayered films: A review. J. Mater. Res..

[19] Michell R.M., Müller A.J. (2016). Confined crystallization of polymeric materials. Prog. Polym. Sci..

[20] Papananou H., Perivolari E., Chrissopoulou K., Anastasiadis S.H. (2018). Tuning polymer crystallinity via the appropriate selection of inorganic nanoadditives. Polymer.

[21] Bollas S., Chrissopoulou K., Andrikopoulos K.S., Voyiatzis G.A., Anastasiadis S.H. (2017). Polymer conformations close to surfaces. Polymers.

[22] Rissanou A.N., Papananou H., Petrakis V.S., Doxastakis M., Andrikopoulos K.S., Voyiatzis G.A., Chrissopoulou K., Harmandaris V., Anastasiadis S.H. (2017). Structural and conformational properties of poly(ethylene oxide)/silica nanocomposites: Effect of confinement. Macromolecules.

[23] Kwiczak-Yiğitbaşı J., Laçin O., Demir M., Erdem Ahan R., Şeker U.O.S., Baytekin B. (2020). A sustainable preparation of catalytically active and antibacterial cellulose metal nanocomposites via ball milling of cellulose. Green Chem..

[24] Alammar A., Park S.-H., Williams C.J., Derby B., Szekely G. (2020). Oil-in-water separation with graphene-based nanocomposite membranes for produced water treatment. J. Membr. Sci..

[25] Elmahdy M.M., Chrissopoulou K., Afratis A., Floudas G., Anastasiadis S.H. (2006). Effect of confinement on polymer segmental motion and ion mobility in PEO/layered silicate nanocomposites. Macromolecules.

[26] Fotiadou S., Chrissopoulou K., Frick B., Anastasiadis S.H. (2010). Structure and Dynamics of Polymer Chains in Hydrophilic Nanocomposites. J. Polym. Sci. Part B Polym. Phys..

[27] Barroso-Bujans F., Cerveny S., Alegría A., Colmenero J. (2013). Chain Length Effects on the Dynamics of Poly(ethylene oxide) Confined in Graphite Oxide: A Broadband Dielectric Spectroscopy Study. Macromolecules.

[28] Androulaki K., Chrissopoulou K., Prevosto D., Labardi M., Anastasiadis S.H. (2015). Dynamics of hyperbranched polymers under confinement: A dielectric relaxation study. ACS Appl. Mater. Interfaces.

[29] Androulaki K., Chrissopoulou K., Prevosto D., Labardi M., Anastasiadis S.H. (2019). Structure and dynamics of Biobased Polyester Nanocomposites. Biomacromolecules.

[30] Elmahdy M.M., Gournis D., Ladavos A., Spanos C., Floudas G. (2020). H-Shaped Copolymer of Polyethylene and Poly(ethylene oxide) under Severe Confinement: Phase State and Dynamics. Langmuir.

[31] Chrissopoulou K., Altintzi I., Anastasiadis S.H., Giannelis E.P., Pitsikalis M., Hadjichristidis N., Theophilou N. (2005). Controlling the Miscibility of Polyethylene/Layered Silicate Nanocomposites by Altering the Polymer/Surface Interactions. Polymer.

[32] Chrissopoulou K., Altintzi I., Andrianaki I., Shemesh R., Retsos H., Giannelis E.P., Anastasiadis S.H. (2008). Understanding and Controlling the Structure of Polypropylene/Layered Silicate Nanocomposites. J. Polym. Sci. Part B Polym. Phys..

[33] Giannelis E.P. (1996). Polymer layered silicate nanocomposites. Adv. Mater..

[34] Ray S.S., Okamoto M. (2003). Polymer/layered silicate nanocomposites: A review from preparation to processing. Prog. Polym. Sci..

[35] Wang J., Hu K.H., Xu Y.F., Hu X.G. (2008). Structural, thermal and tribological properties of intercalated polyoxymethylene/molybdenum disulfide nanocomposites. J. Appl. Polym. Sci..

[36] La Mantia F.P., Scaffaro R., Ceraulo M., Mistretta M.C., Dintcheva N.T., Bota L. (2016). A simple method to interpret the rheological behaviour of intercalated polymer nanocomposites. Comp. Part B Eng..

[37] Habel C., Maiz J., Olmedo-Martínez J.L., López J.V., Breu J., Müller A.J. (2020). Competition between nucleation and confinement in the crystallization of poly(ethylene glycol)/large aspect ratio hectorite nanocomposites. Polymer.

[38] Kawasumi M., Hasegawa N., Kato M., Usuki A., Okada A. (1997). Preparation and mechanical properties of polypropylene-clay hybrids. Macromolecules.

[39] Kim D.H., Fasulo P.D., Rodgers W.R., Paul D.R. (2007). Structure and properties of polypropylene-based nanocomposites: Effect of PP-g-MA to organoclay ratio. Polymer.

[40] Chrissopoulou K., Anastasiadis S.H. (2011). Polyolefin/Layered Silicate Nanocomposites with Functional Compatibilizers. Eur. Polym. J..

[41] Biswas S., Fukushima H., Drzal L.T. (2011). Mechanical and electrical property enhancement in exfoliated graphene nanoplatelet /liquid crystalline polymer nanocomposites. Compos. Part A Appl. Sci. Manuf..

[42] Karevan M., Kalaitzidou K. (2013). Understanding the property enhancement mechanism in exfoliated graphite nanoplatelets reinforced polymer nanocomposites. Compos. Int..

[43] Farahanchi A., Malloy R.A., Sobkowicz M.J. (2018). Extreme shear processing for exfoliating organoclay in nanocomposites with incompatible polymers. Polymer.

[44] Rittigstein P., Priestley R.D., Broadbelt L.J., Torkelson J.M. (2007). Model Polymer Nanocomposites Provide an Understanding of Confinement Effects in Real Nanocomposites. Nat. Mater..

[45] Mondal T., Chandra V., Bhowmick A.K. (2016). Unique method to improve the thermal properties of bisphenol A tetraacrylate by graphite oxide induced space confinement. RSC Adv..

[46] Hu K.S., Kulkarni D.D., Choi I., Tsukruk V.V. (2014). Graphene-polymer nanocomposites for structural and functional applications. Prog. Polym. Sci..

[47] Sun X., Huang C., Wang L., Liang L., Cheng Y., Fei W., Li Y. (2021). Recent Progress in Graphene/Polymer Nanocomposites. Adv. Mater..

[48] Suter J.L., Sinclair R.C., Coveney P.V. (2020). Principles Governing Control of Aggregation and Dispersion of Graphene and Graphene Oxide in Polymer Melts. Adv. Mater..

[49] Sarac E.C., Poudeh L.H., Berktas I., Okan B.S. (2021). Scalable fabrication of high-performance graphene/polyamide 66 nanocomposites with controllable surface chemistry by melt compounding. J. Appl. Polym. Sci..

[50] Chang Y.-W., Lee K.-S., Lee Y.-W., Bang J.H. (2015). Poly(ethylene oxide)/graphene oxide nanocomposites: Structure, properties and shape memory behavior. Polym. Bull..

[51] Larsen R.M., Jensen E.A. (2016). Epoxy–graphite oxide nanocomposites: Mechanical properties. J. Appl. Polym. Sci..

[52] Botlhoko O.J., Ramontja J., Sinha Ray S. (2017). Thermal, mechanical, and rheological properties of graphite and graphene oxide-filled biodegradable polylactide/poly(E-caprolactone) blend composites. J. Appl. Polym. Sci..

[53] Panova T.V., Efimova A.A., Efimov A.V., Berkovich A.K. (2019). Physico-mechanical properties of graphene oxide/poly(vinyl alcohol) composites. Colloid Polym. Sci..

[54] Barroso-Bujans F., Fernandez-Alonso F., Cerveny S., Parker S.F., Alegría A., Colmenero J. (2011). Polymers under extreme two-dimensional confinement: Poly(ethylene oxide) in graphite oxide. Soft Matter.

[55] Barroso-Bujans F., Fernandez-Alonso F., Pomposo J.A., Enciso E., Fierro J.L.G., Colmenero J. (2012). Tunable uptake of poly(ethylene oxide) by graphite-oxide based materials. Carbon.

[56] Barroso-Bujans F., Alegría A., Pomposo J.A., Colmenero J. (2013). Thermal Stability of Polymers Confined in Graphite Oxide. Macromolecules.

[57] Tomalia D.A. (2005). The dendritic state. Mater. Today.

[58] Gao C., Yan D. (2011). Hyperbranched Polymers: Synthesis, Properties, and Applications.

[59] Voit B.I., Lederer A. (2009). Hyperbranched and Highly Branched Polymer Architectures-Synthetic Strategies and Major Characterization Aspects. Chem. Rev..

[60] Gelade E.T.F., Goderis B., de Koster C.G., Meijerink N., van Benthem R., Fokkens R., Nibbering N.M.M., Mortensen K. (2001). Molecular Structure Characterization of Hyperbranched Polyesteramides. Macromolecules.

[61] Zagar E., Zigon M., Podzimek S. (2006). Characterization of Commercial Hyperbranched Aliphatic Polyesters. Polymer.

[62] Dritsas G.S., Karatasos K., Panayiotou C. (2009). Investigation of Thermodynamic Properties of Hyperbranched Aliphatic Polyesters by Inverse Gas Chromatography. J. Chromatogr. A.

[63] Tanis I., Karatasos K. (2009). Local Dynamics and Hydrogen Bonding in Hyperbranched Aliphatic Polyesters. Macromolecules.

[64] Zagar E., Huskic M., Zigon M. (2007). Structure-to-Properties Relation of Aliphatic Hyperbranched Polyesters. Macrom. Chem. Phys..

[65] Malmstrom E., Hult A., Gedde U.W., Liu F., Boyd R.H. (1997). Relaxation Processes in Hyperbranched Polyesters: Effect of Terminal Groups. Polymer.

[66] Zhu P.W., Zheng S., Simon G. (2001). Dielectric Relaxation in a Hyperbranched Polyester with Terminal Hydroxyl Groups: Effects of Generation Number. Macromol. Chem. Phys..

[67] Turky G., Shaaban S.S., Schoenhals A. (2009). Broadband Dielectric Spectroscopy on the Molecular Dynamics in Different Generations of Hyperbranched Polyester. J. Appl.Polym. Sci..

[68] Tanis I., Tragoudaras D., Karatasos K., Anastasiadis S.H. (2009). Molecular Dynamics Simulations of a Hyperbranched Poly(ester amide): Statics, Dynamics, and Hydrogen Bonding. J. Phys. Chem. B.

[69] Fotiadou S., Karageorgaki C., Chrissopoulou K., Karatasos K., Tanis I., Tragoudaras D., Frick B., Anastasiadis S.H. (2013). Structure and Dynamics of Hyperbranched Polymer/Layered Silicate Nanocomposites. Macromolecules.

[70] Moradi L.G., Sari M.G., Ramezanzadeh B. (2020). Polyester-amide hyperbranched polymer as an interfacial modifier for graphene oxide nanosheets: Mechanistic approach in an epoxy nanocomposite coating. Prog. Org. Coat..

[71] Androulaki K., Chrissopoulou K., Labardi M., Anastasiadis S.H. (2021). Behavior of Hyperbranched Polymers: Comparison between Different Layered Nanoadditives. Polymer.

[72] Kremer F., Schönhals A. (2003). Broadband Dielectric Spectroscopy.

[73] Tanis I., Karatasos K., Assimopoulou A.N., Papageorgiou V.P. (2011). Modeling of hyperbranched polyesters as hosts for the multifunctional bioactive agent shikonin. Phys. Chem. Chem. Phys..

[74] Malmstrom E., Liu F., Boyd R.H., Hult A., Gedde U.W. (1994). Relaxation Processes in Hyperbranched Polyesters. Polym Bull..

[75] Ye S., Feng J. (2013). A new insight into the in situ thermal reduction of graphene oxide dispersed in a polymer matrix. Polym. Chem..

[76] Mott F.N., Davis E.A. (1979). Electronic Processes in Noncrystalline Materials.

[77] Capaccioli S., Lucchesi M., Rolla P.A., Ruggeri G. (1998). Dielectric response analysis of a conducting polymer dominated by the hopping charge transport. J. Phys. Condens. Matter..

[78] Ezquerra T.A., Canalda J.C., Sanz A., Linares A. (2014). On the electrical conductivity of PVDF composites with different carbon-based nanoadditives. Colloid Polym. Sci..

