# Supplementary material for: Static and Dynamic Behavior of Polymer/Graphite Oxide Nanocomposites before and after Thermal Reduction
Source: Polymers (Basel). 2021 Mar 25;13(7):1008. doi: 10.3390/polym13071008 (PMC8036730; doi:10.3390/polym13071008)
Supplement: Supplementary file 1 [file polymers-13-01008-s001.pdf]

Supplementary Material

Static and Dynamic Behavior of Polymer / Graphite Oxide Nanocomposites Before and After Thermal Reduction

Kiriaki Chrissopoulou [1,*], Krystalenia Androulaki [1,2], Massimiliano Labardi [3] and Spiros H. Anastasiadis [1,2]

1 Institute of Electronic Structure and Laser, Foundation for Research and Technology-Hellas, N. Plastira 100, 700 13 Heraklion Crete, Greece; krysand@iesl.forth.gr (K.A.); spiros@iesl.forth.gr (S.H.A.)
2 Department of Chemistry, University of Crete, P.O. Box 2208, 710 03 Heraklion Crete, Greece
3 CNR-IPCF, c/o Physics Department, University of Pisa, Largo Pontecorvo 3, 56127 Pisa, Italy; labardi@df.unipi.it
* Correspondence: kiki@iesl.forth.gr; Tel.: +30-281-039-1255

Figure S1 shows the frequency dependence of the imaginary part of the complex permittivity, ε″, as a function of frequency, ω, for the polymer H30 over a wide range of temperatures both below (Fig. S1a) and above (Fig. S1b) the glass transition temperature.

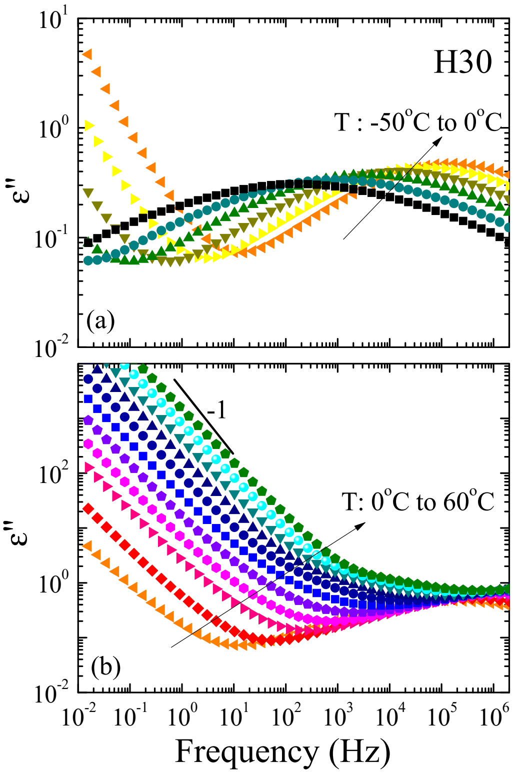

Figure S1: Imaginary part of the complex permittivity, ε″, as a function of frequency, ω, for H30 in the temperature range of -50°C to 0°C (a), 0°C to 60°C (b).

There are no significant differences in the spectra of the three polymers H20, H30 and H40 in the low temperature range (the spectra for H20 were shown in Ref [1] while those for H40 are not shown) and, thus, there is no significant dependence of dynamics on the generation. There is a very broad peak that

covers the whole available frequency window, that may be attributed to motions of the functional groups present in the molecules, like for example hydroxyl rotation or ester reorientation; these are usually named in the literature as β- and γ- secondary relaxation. All three generations of the hyperbranched polyester polyols investigated, exhibit this broad peak at temperatures below 10 °C, which shows very similar characteristics. In the high temperature regime, another relaxation process is evidenced, that can be identified as the segmental α-relaxation. For this process, a dependence on the generation is observed in accordance to their different glass transition temperatures. Indeed, the process appears at lower temperatures and more clearly for H20 [1] and at progressively higher temperatures for H30 and H40. The dynamics of similar hyperbranched polymers has been studied in the past by dielectric spectroscopy. Both the hydroxyl motion and the ester reorientation were evidenced in some cases [2,3], while only one of the two sub T_g motions were found elsewhere [3,4]; these processes have been discussed in detail in Ref. [1].

Figure S2 demonstrates the representative analysis of the spectra of H30 for three temperatures, two below, -70°C (Fig. S2a) and -30°C (Fig. S2b), and one above, 50°C (Fig. S2c) the T_g of the neat H30, utilizing a superposition of the empirical Havriliak-Negami functions together with an additional ionic conductivity contribution at low-frequencies and high temperatures, i.e., with the fitting function of eq. 2

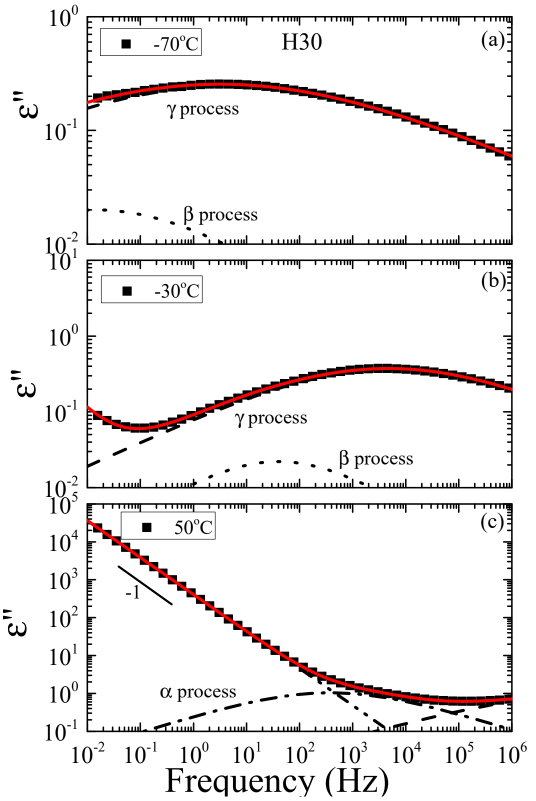

Figure S2: Imaginary part of the dielectric permittivity, ε'', for the H30 at -70°C (a), -30°C (b) and 50°C (c). The processes needed for the deconvolution of the spectra are shown with dashed (γ), dotted (β), dash-dotted (α) and dash-dot-dot (conductivity) lines, whereas the solid lines are the summation of the processes (best fit).

From the analysis, it appears that multiple relaxation processes are necessary to obtain a good fit to the data in all cases. At the lowest temperature, the spectrum is too broad to be fitted with a single relaxation process, which in any case fails to describe satisfactorily the measured data. For the fastest γ-process the β exponent was fixed to 0.9 for H30 (0.8 for H20 [1] and 1.0 for H40), whereas the shape parameter α lies in the interval α: 0.2 − 0.3. The dielectric strength takes values between $\Delta\varepsilon$: 2.8 − 6.7; i.e., they both increase with increasing temperature. At higher temperatures, above the T_g of the neat

polymer, the segmental α-process emerges (dash-dotted line); for this process, the β exponent was fixed to 1.0, whereas the fitted value of the exponent α is around $0.36 - 0.45$ whereas $\Delta\varepsilon$ decreases from 9.3 to 3.7. These values are very similar for all three generations of the Boltorn polymers.

References

1. Androulaki, K.; Chrissopoulou, K.; Prevosto, D.; Labardi, M.; Anastasiadis, S. H. Dynamics of hyperbranched polymers under confinement: A dielectric relaxation study. *ACS Appl. Mater. Interfaces* **2015,** *7*, 12387-12398.
2. Turky, G.; Shaaban, S. S.; Schoenhals, A. Broadband Dielectric Spectroscopy on the Molecular Dynamics in Different Generations of Hyperbranched Polyester. *J. Appl.Polym. Sci.* **2009**, *113*, 2477–2484.
3. Malmstrom, E.; Liu, F.; Boyd, R. H.; Hult, A.; Gedde, U. W. Relaxation Processes in Hyperbranched Polyesters. *Polym Bull.* **1994**, *32*, 679-685.
4. Zhu, P. W.; Zheng, S.; Simon, G. Dielectric Relaxation in a Hyperbranched Polyester with Terminal Hydroxyl Groups: Effects of Generation Number. *Macromol. Chem. Phys.* **2001**, *202*, 3008-3017.
